# Behavioural and Brain Gene Expression Profiling in Pigs during Tail Biting Outbreaks – Evidence of a Tail Biting Resistant Phenotype

**DOI:** 10.1371/journal.pone.0066513

**Published:** 2013-06-18

**Authors:** Emma Brunberg, Per Jensen, Anders Isaksson, Linda J. Keeling

**Affiliations:** 1 Department of Animal Environment and Health, Swedish University of Agricultural Sciences, Uppsala, Sweden; 2 IFM Biology, Linköping University, Linköping, Sweden; 3 Science for Life Laboratory, Department of Medical Sciences, Uppsala University, Uppsala, Sweden; University of Queensland, Australia

## Abstract

Abnormal tail biting behaviour is a major welfare problem for pigs receiving the behaviour, as well as an indication of decreased welfare in the pigs performing it. However, not all pigs in a pen perform or receive tail biting behaviour and it has recently been shown that these ‘neutral’ pigs not only differ in their behaviour, but also in their gene expression compared to performers and receivers of tail biting in the same pen. To investigate whether this difference was linked to the cause or a consequence of them not being involved in the outbreak of tail biting, behaviour and brain gene expression was compared with ‘control’ pigs housed in pens with no tail biting. It was shown that the pigs housed in control pens performed a wider variety of pig-directed abnormal behaviour (belly nosing 0.95±1.59, tail in mouth 0.31±0.60 and ‘other‘ abnormal 1.53±4.26; mean±S.D) compared to the neutral pigs (belly nosing 0.30±0.62, tail in mouth 0.13±0.50 and “other“ abnormal 0.42±1.06). With Affymetrix gene expression arrays, 107 transcripts were identified as differently expressed (p<0.05) between these two categories of pigs. Several of these transcripts had already been shown to be differently expressed in the neutral pigs when they were compared to performers and receivers of tail biting in the same pen in an earlier study. Hence, the different expression of these genes cannot be a consequence of the neutral pigs not being involved in tail biting behaviour, but rather linked to the cause contributing to why they were not involved in tail biting interactions. These neutral pigs seem to have a genetic and behavioural profile that somehow contributes to them being resistant to performing or receiving pig-directed abnormal behaviour, such as tail biting, even when housed in an environment that elicits that behaviour in other pigs.

## Introduction

It is well accepted that both the performers and receivers of injurious abnormal behaviours, such as tail biting in pigs, experience reduced welfare and that the behaviour itself probably develops in response to the environment being unable to satisfy the needs of the animals (reviewed by the European Food Safety Authority, [1]). Even though all pigs at a farm experience a similar environment, especially those housed in the same pen, not all perform or receive the behaviour (Keeling et al., 2004). Therefore, tail biting research has moved towards investigating individual characteristics of pigs, with a view to obtaining information about internal factors contributing to the development of the behaviour. These studies have led to new knowledge about differences in behaviour (e.g. [2], [3], [4]) and production traits (e.g. [4], [5]) associated with tail biting. But since tail biting can still be difficult to prevent, even with proper housing, up to 90% of the pigs within the European Union (EU) have docked tails [1]. The ban on routine tail docking within the EU (EU Directive 91/630 EEC) makes it even more important to understand the biological mechanisms underlying tail biting behaviour to be able to prevent it.

In a previous study [6], brain gene expression was investigated in pigs housed in the same pen, but performing and receiving different amounts of tail biting behaviour (i.e. tail biters, receivers and neutral animals). Differences were found and it was suggested that these could contribute towards identifying the mechanisms underlying tail biting behaviour. However, it was also noted that while only three genes were differently expressed when comparing performers and receivers, 37 and 135 genes were differently expressed when comparing neutral pigs, not performing or receiving tail bites, to performers and receivers respectively. Moreover, the expression of 19 genes was different in both tail biters and receivers compared to the neutral pigs and it was therefore suggested that rather than focussing on why some pigs become performers or receivers of tail biting, it might be worthwhile to focus on why some pigs become neither. A closer investigation of these 19 genes for which neutral pigs had a different expression pattern, may provide information about why such pigs are not involved in tail biting behaviour and, ultimately, how to prevent the occurrence of this behaviour in practice. The fact that genes with possible functions in production traits, explorative and social behaviour were differently expressed corresponds well with earlier studies (e.g. [5], [7], [8]).

As with all gene expression studies it is difficult to determine whether the differently expressed genes are related to the cause or a consequence of the trait of interest. The expression of the 19 genes may have predisposed tail biters and receivers to be involved in tail biting compared to the neutral pigs (alternatively neutral pigs *not* to be involved). Another option is that the different expression pattern was an effect of them performing and receiving tail bites (alternatively neutral pigs *not* performing or receiving them). The present study aimed to investigate this question. To address this, the current study compares previous reported differently expressed genes in pigs housed in a tail biting pen, with gene expression in a new category of pigs, namely control pigs not involved in tail biting behaviour and housed in a pen without an outbreak of tail biting. Being able to exclude that the differently expressed genes were a consequence of the difference in tail biting behaviour, would provide the first support for the hypothesis that differences in gene expression contribute to neutral pigs being resistant to becoming involved in tail biting and support the suggestion that this should be the focus of future studies.

Since neutral and control pigs are housed in different pens with a different tail biting situation, gene expression differences due to environment are expected. Higher activity levels in tail biting pens has also been shown [9]. It is well known that housing and social interactions affect the stress level of an animal [10] and a tail biting outbreak probably leads to a stressful environment. It is also known that gene expression is very much influenced by environmental factors, such as stress. For example, it has been shown that only 15 minutes of social isolation of piglets led to a different gene expression in the prefrontal cortex [11]. However, if the 19 genes that were differently expressed when comparing neutrals to performers and receivers are also differently expressed when comparing neutrals and controls, then the expression differences of these specific genes is neither due to differences in the two pen environments nor a direct consequence of the ongoing outbreak of tail biting. Instead, it would imply that they are related to the cause of tail biting and so their functions are of special interest in order to identify why some pigs do not become involved in an outbreak of tail biting.

## Materials and Methods

### Animals and Housing

The present study was performed in Finland on a farm with a history of tail biting problems, producing fattening pigs (Finnish Yorkshire x Finnish landrace x Duroc (x Hampshire)) from approximately 25 kg to slaughter. In accordance with the animal welfare legislation in Finland, no pigs had docked tails and the males were castrated during the first week of age. Environment and housing has been described previously in detail [2] and [6]. The study was approved by the ethical board at Helsinki University and all efforts were made to minimize stress and suffering in the animals.

### Behavioural Observations

The study was performed during four periods in 2009; May, June-July, September and October and the stables were emptied between the second and the third periods, due to the all-in-all-out production system. Hence, all pigs housed at the farm during the same observation period were of the same age and were between 10 and 21 weeks of age during the observations.

To be able to select pigs which varied in their tail biting behaviour, observations were carried out in pens with signs of an ongoing tail biting outbreak (mainly based on tail status or short behavioural observations) as well as in pens with no apparent tail biting problems. In total, 742 pigs in 58 pens were individually marked with colour spray and performed and received tail and ear biting, belly nosing, mounting, as well as any bar biting or other abnormal behaviours were recorded during 30 minutes using continuous all occurrence sampling and the same ethogram as in [2]. The key behaviour tail biting was defined as when one pig was biting and chewing the tail of another pig. If the performer let go of the tail to immediately bite again, this was regarded as a new registration. The identity of all performer and receiver pigs was noted. Observations were repeated later the same day or the day after, giving a total observation time of 60 minutes out of which 30 minutes were performed in the morning and the other 30 minutes in the afternoon. Following these observations, it was decided if the pen was a so-called tail biting pen (ongoing outbreak of the behaviour, including one or more tail biters) or a control pen (no evidence of an ongoing outbreak of tail biting). If it was not possible to categorise the pen after 60 minutes of observation (i.e. the pigs were inactive during the observations) the tail biting pen and its matched control pen were observed for an additional 30 or 60 minutes.

When a matched pair of pens consisting of one tail biting and one control pen in the same building was selected, candidate pigs were selected that were neither performers nor receivers of tail biting. The criteria were that the pig did not perform any tail bites and did not receive more than one tail bite during 60 minutes of observation. Following the same ethogram as in previous observations, the selected pigs were then individually observed for 2 hours (8×15 minutes) during one or two days to confirm that the classification as either a neutral pig, (defined as a pig housed in a tail biting pen but without being involved in the tail biting behaviour, i.e. being neutral to the tail biting outbreak), or a control pig, (defined as a pig housed in a control pen and hence not involved in tail biting behaviour) was correct. Due to the tail status of the pigs, it was not possible for the observer to be blind to the pen. Therefore, to decrease the possibility of biased data, at least two independent observers performed the observations for each pen/individual.

### Euthanisation and Tissue Sampling

After the observations, 6 pairs of one neutral pig and one control pig, each matched for age, sex, building, feeding system and pen size, were selected for tissue sampling. To minimize stressing these 12 individuals before euthanisation, the pigs were sedated in their home pens with an intramuscular injection of midazolam (0.5 ml/kg), directly followed by an intramuscular injection of butorphanol (0.20 mg/kg) and ketamine (10 mg/kg). The sedated pig was moved to an adjacent room where it was euthanized with an intracardial injection of pentobarbital (approximately 20 mg/kg), while still in anaesthesia. When no corneal reflex was shown, the brain was removed and the hypothalamus and prefrontal cortex were dissected. Liquid nitrogen was used to freeze the tissues, which were transported on dry ice and preserved in −80°C.

### RNA Extraction and Microarrays

The left hypothalamus and left side of the prefrontal cortex were used in further analyses. The hypothalamus was used based on its involvement in stress mechanisms, energy homeostasis and feeding behaviour and also since we earlier have used the hypothalamus to explore gene expression in feather pecking hens [12]. The prefrontal cortex plays a role in cognitive functions and seems to be involved in many psychological diseases. To get representative samples of these two brain areas, liquid nitrogen and mortars were used to crush the tissues prior to homogenization in Qiazol. After isolating RNA with Qiagens RNeasy lipid tissue mini kit (Applied Biosystems, Valencia, CA), a ND-1000 spectrophotometer (NanoDrop Technologies, Wilmington, DE) and Agilent 2100 Bioanalyzer (Agilent Technologies Inc, Palo Alto, CA) were used to control concentration and quality.

For the gene expression arrays, samples from six matched pairs were chosen. During the hybridization, washing and scanning procedure, the GeneChip® 3′ IVT Express Kit Manual (PN 702646 Rev1) and the Wash, Stain and Scan Manual (PN 702731 Rev2, Affymetrix Inc., Santa Clara, CA) were followed. Shortly, biotinylated fragmented cRNA was prepared from 250 nanograms total RNA. This was then hybridized onto Affymetrix GeneChip® porcine expression arrays in 45°C, rotated at 60 rpm for 16 hours. A Fluidics Station 450 and GeneChip® Scanner 3000 7 G were used to wash and stain the arrays.

### Data Analysis

Due to technical and quality problems, three matched pairs had to be removed from the analysis of the microarray data from the hypothalamus and two from the prefrontal cortex, leaving three matched pairs to be used in the analysis of the hypothalamus and four from the prefrontal cortex.

#### Behavioural data

To obtain representative behaviour profiles to compare with the genetic profiles obtained in the microarrays, behaviour of neutral pigs housed in tail biting pens and control pigs housed in control pens was compared. The behaviour of 60 neutral pigs, housed in the same tail biting pens as the six selected neutral pigs, and 99 control pigs, housed in the same control pens as the finally selected 6 control pigs, was analysed. For the pigs in the one pair of pens that were observed for more than 60 minutes, the observations for the last two 30-minute observation periods were used, as the selection of pigs for tissue sampling were based on those observation periods. The analyses of behaviour were performed using the procedures RANK and MIXED in SAS version 9.2 (SAS institute, Inc. Cory, NC). For each behaviour, the pigs were ranked using Blom normal rank scores according to the frequency they performed and received each particular behaviour. When comparing performed and received behaviours between neutral and control pigs, a model including the fixed effects of category, sex, stable and group-pen nested within stable, and the random effect of pair was used.

#### Microarray data

All gene expression data are deposited to ArrayExpress with the accession number E-MEXP-3643. For normalization of the raw gene expression data, the multi-array average (RMA) method [13], [14] was used in the software Expression Console (provided by Affymetrix, www.affymetrix.com). An empirical Bayes moderated t-test [15] using the limma package was used when searching for differences between the pig categories. Using the method of Benjamini and Hochberg [16] the p-values were adjusted to correct for multiple testing. This was performed in the statistical computing language R (http://www.r-project.org) with packages available from the Bioconductor project (www.bioconductor.org).

#### Enrichment analysis

To explore enriched gene ontology (GO) and Kyoto Encyclopedia of Genes and Genomes (KEGG) pathways among the differently expressed genes in the hypothalamus, DAVID Bioinformatics Resources 6.7 (http://david.abcc.ncifcrf.gov) was used. Default settings were used, but to maximize the amount of information, the probe set IDs for the corresponding human array were used and hence the human genome used as background. The functional annotation chart function was used and the criteria for a term to be regarded as interesting were that the fold enrichment was above 1.5, the number of genes more than three [17] and the difference significant (p<0.05). Significance values that are not corrected for false discovery rate (i.e. Bonferroni and Benjamini) were used since corrected values could in an early stage lead to less sensitivity when interpreting the results [17].

## Results

### Behaviour

**Figure 1:**
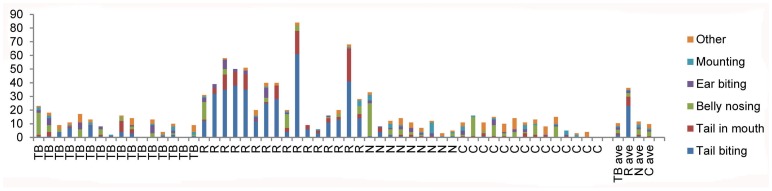
Performed and received abnormal behaviours for all pig categories. The number of performed (Figure A) and received (B) tail biting, tail in mouth, belly nosing, ear biting, mounting, bar biting and other for all the 56 pigs (tail biters; TB, receivers; R, neutrals, N, controls, C) that were sampled in the present study as well as in [6]. The four bars to the right represent the average (Ave) performed behaviours for each pig category. Only a few of the animals included in this figure were later used in the microarrays.

When comparing all neutral pigs in the 6 tail biting pens (n = 60) with all control pigs in the 6 control pens (n = 99) several behavioural differences were found. Neutral pigs performed less tail in mouth, belly nosing and ‘other abnormal’ behaviour, but more bar biting than control pigs. Neutral pigs also received less ‘other abnormal’ behaviour directed to them and tended to receive less belly nosing, although they were mounted more often than control pigs. Means and p-values are presented in table 1. None of the individuals selected for the microarrays (four females and four males) performed any tail bites, but one of the neutral pigs received one. The behaviour of all pigs (n = 56) that were sampled both in the present study and [6] (both pen and individual observations) is shown in figure 1.

**Table 1:** Performed and received abnormal behaviours (mean±S.D.) in neutral pigs (N) housed in tail biting pens (n = 60) compared to control pigs (C) housed in control pens (n = 99).

		Performed				Received			
	D.F	N	C	P	T	N	C	P	T
Tail in mouth	146	0.13±0.50	0.31±0.60	0.01	2.52	0.30±0.59	0.38±0.82	n.s.	0.24
Belly nosing	146	0.30±0.62	0.95±1.59	<0.01	2.64	0.42±1.46	1.06±2.60	0.06	1.89
Ear biting	146	0.30±0.89	0.46±1.38	n.s.	1.14	0.52±1.05	0.54±1.22	n.s.	−0.25
Mounting	146	0.30±1.01	0.19±0.70	n.s.	−1.19	0.63±1.40	0.18±0.48	<0.01	−2.75
Bar biting	146	0.52±1.43	0.11±0.35	0.04	−2.11				
Other abnormal	146	0.42±1.06	1.53±4.26	<0.01	3.42	0.97±1.00	1.34±1.92	0.02	2.42

### Gene Expression and Enrichment Analysis

In total, 107 transcripts were differently expressed (p<0.05) in the hypothalamus and 10 in the prefrontal cortex when comparing the neutral pigs (housed in tail biting pens) with the controls (housed in control pens with no tail biting). Of these, four were differently expressed in both brain areas. Moreover, when also comparing gene expression in the new category of control pigs with tail biters and receivers from a previous study [6], nine transcripts in the hypothalamus and one in the prefrontal cortex were differently expressed in tail biters vs. controls and three and four transcripts were differently expressed in the hypothalamus and prefrontal cortex, respectively, when comparing receivers and controls. One of the genes was differently expressed both when comparing tail biters and controls as well as neutrals compared to controls.

**Figure 2:**
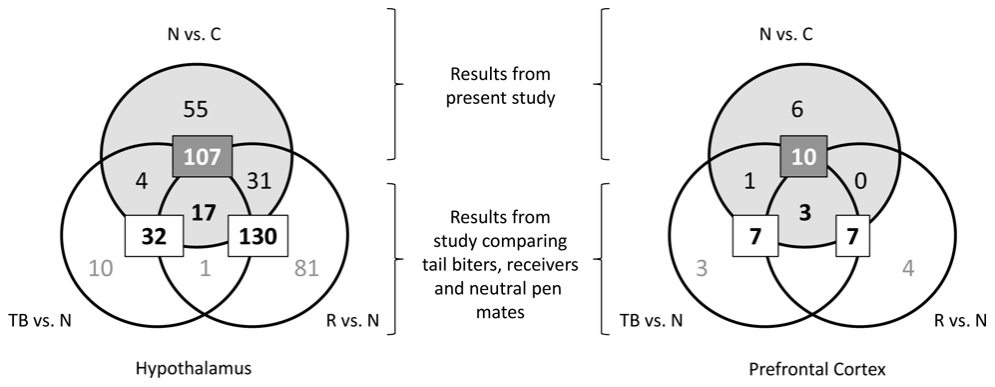
Number of differently expressed genes in the pig categories. Comparison of the differently expressed genes in the present study comparing neutral pigs in a tail biting pen with control pigs in a non-tail biting pen (Grey circle and box) with the gene lists from an earlier study comparing tail biters (TB), receivers (R) and neutral pigs (N) ([6], white circles and boxes). The boxes indicate the total number of differently expressed transcripts in the three comparisons. Out of these, 17 transcripts in the hypothalamus and 3 in the prefrontal cortex were differently expressed in all three comparisons, suggesting that for many of the genes, the N pigs are different from all other categories.

**Figure 3:**
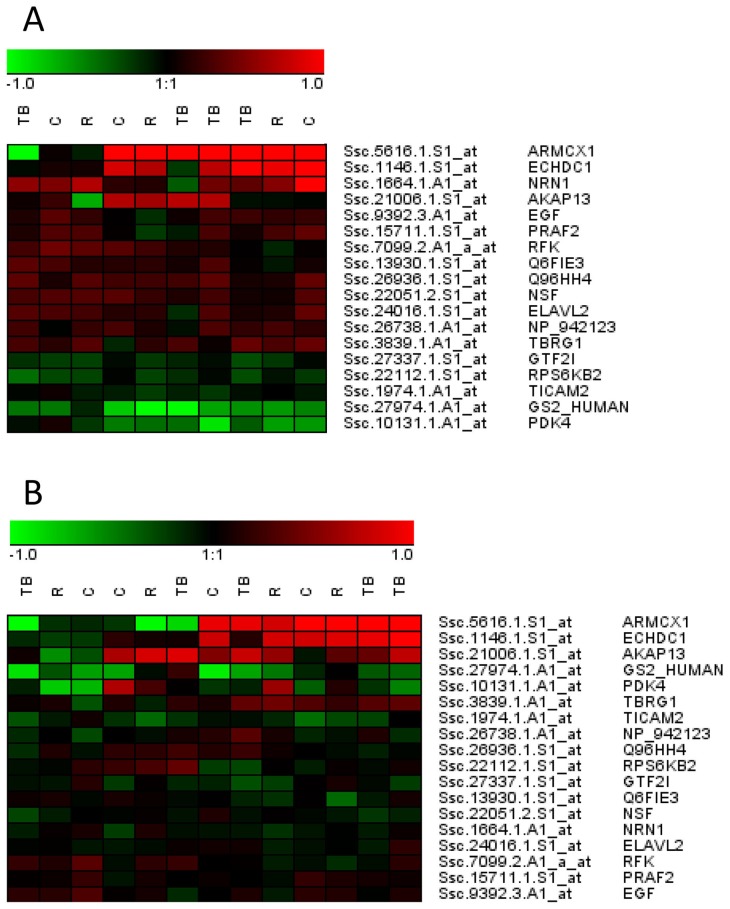
Heat maps containing genes that were differently expressed in neutral pigs compared to all other pig categories. Each spot in the figure represent the expression difference in the individual tail biters (TB), receivers (R) and controls (C) compared with the neutral pig in the same batch, in hypothalamus (Figure A) and prefrontal cortex (B). A green colour means that the gene is less expressed in the individual compared to the corresponding neutral pig, while a red colour means that it is more expressed.

From the earlier study [6] it was already known which genes were differently expressed between neutral pigs and those involved in the tail biting outbreak as performers or receivers. Out of the 117 differently expressed transcripts in the present study comparing neutral and control pigs, 56 were differently expressed also in one or several of the possible comparisons in that previous study. Seventeen of the genes in the hypothalamus and three in the prefrontal cortex were differently expressed in the neutral pigs compared to all other categories of pigs (Figure 2). One was also differently expressed in receivers compared to both neutrals in the study by Brunberg et al. [6] and the control pigs in the present study. The difference in the number of genes that were differently expressed in the hypothalamus and prefrontal cortex is illustrated in Figure 3. The heat map is visualising the relative gene expression when comparing the tail biters, receivers and controls with their matched neutral pig.

Table 2 lists the differently expressed genes that were significant *both* when comparing the neutral pigs to the controls in the current study and in the neutral pigs compared to tail biters and receivers in the earlier study. Two of these were significant in both hypothalamus and prefrontal cortex. The 61 remaining differently expressed transcripts were so in only the neutral vs. control comparison. All differently expressed transcripts are listed in Dataset S1.

**Table 2:** Affymetrix probe set IDs, gene names, brain area, log fold changes (LFC) and p-values for the transcripts that were differently expressed in neutral pigs compared to control pigs and that also were reported to be differently expressed when comparing the neutral pigs with pen mates performing and receiving tail biting [6].

Probe Set ID	Gene	Brain area	LFC	P
Ssc.5616.1.S1_at	*ARMCX1*	Hypothalamus	−1.84	0.0000
Ssc.1146.1.S1_at	*ECHDC1*	Prefrontal cortex	−0.64	0.0002
Ssc.1146.1.S1_at	*ECHDC1*	Hypothalamus	−0.86	0.0011
Ssc.10131.1.A1_at	*PDK4*	Hypothalamus	0.56	0.0014
Ssc.7099.2.A1_a_at	*RFK*	Hypothalamus	−0.28	0.0014
Ssc.27974.1.A1_at	*GS2_HUMAN*	Hypothalamus	0.72	0.0051
Ssc.24016.1.S1_at	*ELAVL2*	Hypothalamus	−0.29	0.0053
Ssc.1664.1.A1_at	*NRN1*	Hypothalamus	−0.45	0.0076
Ssc.21006.1.S1_at	*AKAP13*	Hypothalamus	−0.45	0.0088
Ssc.22051.2.S1_at	*NSF*	Hypothalamus	−0.25	0.0088
Ssc.3839.1.A1_at	*TBRG1*	Hypothalamus	−0.31	0.0088
Ssc.15711.1.S1_at	*PRAF2*	Hypothalamus	−0.25	0.0136
Ssc.13930.1.S1_at	*Q6FIE3*	Hypothalamus	−0.23	0.0158
Ssc.1974.1.A1_at	*TICAM2*	Prefrontal cortex	0.22	0.0250
Ssc.22112.1.S1_at	*RPS6KB2*	Hypothalamus	0.26	0.0269
Ssc.26738.1.A1_at	*NP_942123*	Hypothalamus	−0.23	0.0295
Ssc.9392.3.A1_at	*EGF*	Hypothalamus	−0.25	0.0296
Ssc.27337.1.S1_at	*GTF2I*	Hypothalamus	0.26	0.0365
Ssc.26936.1.S1_at	*Q96HH4*	Hypothalamus	−0.31	0.0403
Ssc.21006.1.S1_at	*AKAP13*	Prefrontal cortex	−0.46	0.0490

According to the criteria for the enrichment analysis, 54 GO terms were significant, but many of the terms were also reported to be significant in [6]. Table 3 lists the 18 terms from the enrichment analysis that were significant in *both* this current and the earlier study. All significant terms are listed in Dataset S2.

**Table 3:** Gene ontology terms (P -value and fold enrichment) that were significant in the enrichment analysis in the gene list from the neutral vs. control pigs comparison.

Category	Term	Count	P	FE
Biological process	GO:0030030∼cell projection organization	8	0.0049	3.77
Biological process	GO:0030182∼neuron differentiation	7	0.0384	2.77
Biological process	GO:0031175∼neuron projection development	7	0.0033	4.74
Biological process	GO:0046907∼intracellular transport	11	0.0040	2.90
Biological process	GO:0048666∼neuron development	7	0.0127	3.58
Biological process	GO:0048812∼neuron projection morphogenesis	6	0.0073	4.89

Only those terms that were reported to be differently expressed also when comparing the neutral pigs with pen mates performing and receiving tail biting [6] are listed.

## Discussion

The results from this study indicate that pigs not participating in tail biting as performers or receivers, differ in the type of abnormal behaviour they perform and also in brain gene expression, depending on if they are housed in a pen with an ongoing tail biting outbreak (neutral pigs in tail biting pens) or in a nearby pen selected for lack of tail biting (control pigs in control pens).

Even if the pigs in the control pen did not develop tail biting behaviour, they performed more pig-directed abnormal behaviours compared to the neutral pigs that were housed in pens in which tail biting was occurring. Control pigs performed a higher frequency of tail in mouth, belly nosing and ‘other abnormal’ behaviours compared to the neutral pigs, which instead performed more bar biting. The control pigs had more ‘other abnormal’ behaviour and tended to have more belly nosing directed towards them. Neutral pigs received more mounting, but this is not necessarily an abnormal behaviour. Hence, the neutral pigs were not only less involved in tail biting behaviour compared to their pen mates that were performers and receivers, but they also performed and received less other pig-directed abnormal behaviours compared to control pigs. Bar biting was the only behaviour that the neutral pigs performed more of compared to the control pigs, and it was also the only behaviour that was not pig-directed. It could be speculated that nearly all pigs in the environment on this farm were motivated to perform abnormal behaviour, but since neutral pigs are less pig-directed they directed their behaviour towards pen fittings and allowed less pig-directed behaviour to be directed towards them. We therefore suggest that the mechanism by which these pigs were resistant to the tail biting was by them being less social in their behaviour. The pigs were not observed after the specified observation period, so it is not possible to know whether or not some of the pigs in the control pens developed tail biting behaviour later, i.e. if the pig-directed behaviours performed by the control pigs later develop into tail biting or other types of pig-directed abnormal behaviours (such as tail biting). A more detailed data analysis of the behaviour of all 742 pigs included in study has been published previously [2].

While only 10 and 7 genes respectively were differently expressed in the control pigs in the control pens compared to tail biter and receiver pigs in the tail biting pens selected in the earlier study by Brunberg et al. [6], 107 were differently expressed in comparison with the neutral pigs housed in tail biting pens. This is in line with the gene expression data in our previous study comparing tail biters and receivers with control pigs in which very few gene expression differences were found when comparing tail biters with receivers, in contrast to when these two categories were compared to neutral pigs [6]. Hence, it seems like the neutral pigs differ most in gene expression compared to the other three categories of pigs, while these other three categories are relatively similar to each other, despite the difference in performed and received tail bites. Interestingly, 18 out of the 19 genes that were differently expressed when comparing the neutral pigs to both tail biters and receivers in the earlier mentioned study [6] were also differently expressed when comparing them to the control pigs. It can therefore be excluded that the different expression of these genes is a consequence of the neutrals not being involved in tail biting behaviour, since they differed also compared to the control pigs not involved as performers or receivers. It can further be excluded that the expression of these genes is a consequence of the different environment for the neutral and control pigs, since the tail biters and receivers also differed from the neutral pigs in the expression of these genes and these pigs all experienced the same pen environment. We therefore suggest that the different expression of these 18 genes contribute to the tail biting resistant phenotype of the neutral pigs and that the functions of these genes may help identify biological mechanisms important for the development of tail biting.

Of the 18 genes, 17 were differently expressed in the hypothalamus and only three in the prefrontal cortex. This difference between brain areas is also clear when visualizing the genes that were differently expressed in the neutral pigs compared to all other categories in a heat map (Fig. 3). This difference in gene expression between the two brain areas could indicate that pathways that involve the hypothalamus are more important for the development of tail biting than pathways involving the prefrontal cortex. The hypothalamus is known to being highly involved in stress and feeding behaviour, two mechanisms that are also thought to be important in tail biting behaviour. The dissection methods can also have influenced the difference between the two areas. The hypothalamus is a smaller and more distinct area when performing the dissections like it was done in this project. That the prefrontal cortex is larger and perhaps less specific could contribute to diluting possible expression differences in that area.

It should be noted that the differences in gene expression may not be the direct cause of the difference in behaviour. The difference in gene expression may be caused by another factor (individual or environmental) that not only influences the expression of certain genes, but also influences tail biting behaviour. However, the functions of the genes that are differently expressed in the neutral pigs are then important clues to reveal why these animals are neutral in a tail biting outbreak. A limitation of the study is the small number of animals, hence more research in this area including more animals from different farms would be of benefit.

Focussing on the function of genes differently expressed in neutral pigs compared to all other pig categories (performers and receivers housed in the same tail biting pens as the neutral pigs and controls housed in control pens without an outbreak of tail biting), and the knowledge it gives about why neutral pigs are resistant to this injurious abnormal behaviour, is a new approach to understanding tail biting behaviour. The functions of some of these genes were discussed in [6] and it was concluded that the different expression of *PDK4* (with effects on fat content in pigs [18]), *GTF2I* (with possible effects on social behaviour in mice [19] and humans [20]) and *EGF* (with possible effects on novelty seeking in humans [21]) can easily be linked to tail biting behaviour. Interestingly, mice with a lower expression of *GTF2I* due to a heterozygous knock-out, showed more interest in unfamiliar mice, but were not more interested in a novel object compared to a more familiar object. This fits very well with our suggestion that neutral pigs (with a higher mRNA level of *GTF2I*) were less interested in other pigs and directed their abnormal behaviour to pen fittings. That production traits, such as a lower back fat thickness, are associated with tail biting is known from earlier studies [5], [7], and the conclusion from these studies has been that the selection for higher production has created pigs that are more prone to perform tail biting. In our study, the control pigs were not involved in tail biting behaviour, but still had a lower expression of *PDK4* compared to neutral pigs. Therefore, our results, together with the previously presented data [6], instead indicate that this selection predisposed pigs to be involved in *any* pig-directed abnormal behaviours as performers and/or receivers. A possible link between production traits and both performing and receiving not only tail biting, but also other pig-directed abnormal behaviours, has to our knowledge not yet been investigated. Our findings suggest that this would be a worthwhile future study. Also in the enrichment analysis, many of the terms that were enriched in the neutral vs. control analysis were the same as the ones enriched in the neutral pigs compared to tail biters and receivers. These gene ontology terms all seem to be linked to neuron development.

Despite the rather large number of studies that have investigated the environmental factors influencing tail biting behaviour, tail biting is still a rather common problem in pig production. It has therefore been proposed to identify and compare the individuals performing the behaviour with those not developing the behaviour [22]. That so many of the genes were differently expressed in neutral pigs compared to the control pigs, shows how important it is to choose control animals from the actual problem pen when making these comparisons. Both external and internal factors affect the development of tail biting and it was not known if any environmental factor triggering tail biting had been present in the tail biting pens compared to control pens, or if the difference between levels of tail biting in the two pen types was due to individual differences among the pigs. Hence, it was not possible to know the true phenotype of a control animal in a pen in which tail biting behaviour has not (yet) developed, i.e. if the pig would be a performer, receiver or neutral during a tail biting outbreak. That so few gene expression differences were found when comparing the controls to tail biters and receivers, may indicate that these categories were rather similar and that the control animals in control pens were probably a mixture of possible performers, receivers and neutral pigs even if this phenotype was not shown. That other abnormal behaviours are linked to tail biting [2], [4] and because pigs in the control pens performed higher levels of other abnormal behaviours, further supports that they are not optimal controls. Therefore, even the performance of other pig-directed behaviours should be taken into account when selecting appropriate control pigs to be compared to individuals performing and receiving tail biting.

In total, there were 61 differently expressed genes (54 in hypothalamus and 5 in prefrontal cortex and one in both areas) that were unique in the neutral vs. control pig comparison (and hence not reported as differently expressed in the earlier study comparing neutral pigs with performers and receivers of tail biting). These may be considered genes that could be affected by differences in the pen environments that the neutral and control pigs experienced. These could be differences in the physical environment, such as ventilation and location in the stable between control and tail biting pens, which contributed to the difference in tail biting behaviour in the two pen types. They could also be differences in the social environment, since it is likely that neutral pigs were affected by the ongoing tail biting outbreak involving the other pigs in their pen. The gene ontology terms unique for the neutral vs. control comparison indicated that genes with roles in different homeostatic processes were enriched. Since stress is traditionally said to be a response to when the homeostasis of an organism is threatened [23], this fits well into the hypothesis that the environment of being housed in a tail biting pen is stressful to the individual pig, irrespective of whether it is involved in the tail biting or not. It is of particular interest that genes involved in cellular calcium homeostasis were enriched, since Holmgren et al. (2000) found a strong tendency for serum calcium to be higher in pigs in control pens compared with pigs in tail biting pens. The same tendency was even found when comparing serum calcium levels in biters, receivers and neutral pigs within a tail biting pen. Among the enriched genes, with functions in cellular calcium ion homeostasis, is *GHRL* which encodes ghrelin-obestatin preproprotein, generating ghrelin and obestatin. Ghrelin is an appetite regulating hormone increased during fasting. It reduces energy expenditure and has effects on obesity (reviewed by [24]). In rodents, plasma ghrelin levels are increased during emotional and physical stress [25], [26], [27], and mice which were exposed to ghrelin prenatally were less explorative in an open field test [27]. This may fit well with *GHRL* mRNA being more abundant in neutral pigs (housed in the presumably more stressful tail biting pens) compared to controls. *COMP,* which encodes cartilage oligomeric protein, was also more expressed in neutral pigs compared to controls. *COMP* mRNA was one of the genes more expressed in feather pecking hens compared to their receivers [12], which is interesting since feather pecking and tail biting in pigs are two abnormal behaviours with several similarities. The protein is a noncollagenous extracellular matrix protein, but its function in the brain to our knowledge has not been studied.

In summary, behavioural and gene expression data from the present study indicate that neutral pigs housed in tail biting pens differ in behaviour and brain gene expression, not only compared to their performer and receiver pen mates, as suggested in an earlier study, but also to other control pigs housed in control pens without a tail biting outbreak. It was further concluded that the genes differently expressed in these neutral pigs were associated with the cause, rather than the consequence, of them not performing and receiving tail biting. A major difference was that these neutral pigs performed less pig-directed abnormal behaviour than the other categories of pigs, which fits well with differences in the expression of genes linked to social and exploration behaviour. Moreover, the results also suggest that selection on production traits influenced tail biters, receivers and controls to be more involved in pig-directed abnormal behaviour. In combination, these results imply that, given similar physical environmental conditions, whether an individual becomes a tail biter, has its tail bitten or remains neutral to a tail biting outbreak, is related to how much its behaviour is targeted towards pen mates. Neutral pigs are less pig-directed in their behaviour. It is proposed that this knowledge could lead to a new approach to reducing the severe and wide spread problem of tail biting in commercial pig production.

## Supporting Information

Dataset S1 **All differently expressed transcripts with log fold changes and p-values.**
(XLS)

Dataset S2 **All significant enrichment terms.**
(XLS)
